# Williams Syndrome with a “Twist”

**DOI:** 10.1155/2010/726845

**Published:** 2010-06-16

**Authors:** Despoina Maritsi, Lydia Kossiva, George Vartzelis

**Affiliations:** ^1^Rheumatology Department, Great Ormond Street Hospital for Children NHS Trust, Great Ormond Street, London WC1H 3JH, UK; ^2^Departement of Academic Pediatrics, “P. & A. Kyriakou” Children's Hospital, Athens 115-21, Greece

## Abstract

Williams syndrome is a rare genetic condition with multisystemic involvement, caused by a microscopic deletion in the chromosome band 7q11.23. We describe the first case of a toddler with Williams syndrome who developed Benign Paroxysmal Torticollis (BPT), a benign dystonic disorder of unknown aetiology.

## 1. Case Report

Our patient is a 14-month-old girl who presented with recurrent episodes of head tilting, vomiting, and drowsiness. The episodes started at the age of 8 months, occurring approximately twice every month and lasting from two hours up to 4 days. Imaging studies by means of brain and neck MRI, as well as electroencephalography during one of the episodes, revealed no abnormalities. Ophthalmology and ENT assessment were also unremarkable. In view of the absence of any obvious underlying pathology and the typical clinical presentation, a diagnosis of BPT was made. Developmental history was significant for moderate motor and social delay, and there was a strong family history of migraine, but no other family history of note. During the physical examination the patient was found to have poor growth (weight, head circumference, and height <3rd centile), moderate dysmorphic features, and a grade-3 systolic murmur heard over the second intercostal space on the left. Cardiac ultrasound confirmed the presence of a moderate supravalvular pulmonary stenosis, while the electrocardiogram was normal. In view of these findings the possibility of Williams syndrome was raised. Subsequently the patient was tested with Fluorescent In Situ Hybridization (FISH) which revealed a microscopic 7q11.23 deletion, thus confirming the diagnosis of Williams syndrome. Although the episodes of torticollis improved with regards to frequency and intensity, they were still present at the age of 20 months.

## 2. Discussion

BPT is a benign, self-limiting disorder of unknown aetiology. Hallmark of this condition is the recurrent episodes of head tilting which last from a few hours up to a number of days. The head tilting results from dystonic spasm of the sternocleidomastoid muscle and can alternate from side to side [1]. Frequent accompaniments are vomiting, nystagmus, pallor, and irritability. Although the aetiology of BPT remains unknown, it has been speculated that the underlying disorder is probably a channelopathy. A number of BPT patients have been described with a family history of paroxysmal disorders such as hemiplegic migraine and episodic ataxia. In some of these cases mutations have been found of the CACNA1A gene, encoding the alpha1A subunit of the voltage-dependent calcium channel or the ATP1A2 gene, encoding a catalytic subunit of a sodium-potassium ATPase [2–4]. However these genes are located in chromosomes 19p13 and 1q23, respectively, remotely from the area of the genetic defect in Williams syndrome (7q11.23). 

The latter is a sporadic genetic disorder with a prevalence of 1 in 20000 in the general population. A microscopic deletion in the band 7q11.23 can be found in 95% of the cases. The clinical manifestations include dysmorphic features, cardiovascular anomalies (supravalvular aortic stenosis and peripheral pulmonary stenosis), hypercalcaemia, and a characteristic behavioral profile. 

To our knowledge Williams syndrome is not known to be associated with BPT. Furthermore the genetic defects of various paroxysmal movement disorders like episodic ataxia or dystonia are not proximal to the band 7q11.23. Thus the occurrence of these two rare conditions in our patient is most likely fortuitous. It is interesting however that patients with Williams carry an increased risk for sudden death which is estimated to be 25 to 100-fold higher than aged matched individuals [5–7]. The causative factors are thought to be related to the associated cardiac abnormalities, namely, the aortic and pulmonary stenosis as well as to myocardial ischemia secondary to coronary insufficiency. However all cases of unexpected death can not be explained by the above abnormalities, and in these individuals other causes, like malignant cardiac arrhythmias could be responsible [5, 8]. Certain types of channelopathies are known to cause severe arrhythmias and sudden death. The elevated calcium levels exhibited by individuals with Williams could complicate further the electrographic diagnosis in such patients. In the case of a child with long-QT syndrome and Williams described by Czosek and Berul, the conduction abnormality was identified on the electrocardiogram only after the patient's hypercalcaemia was corrected [9]. The possibility of an unknown channelopathy with cardiac as well as cerebral manifestations could offer a theoretical model explaining the cases of sudden death in patients with Williams syndrome as well as the BPT, seen in our patient.

## 3. Conclusion

We presented the first case of a child with Williams syndrome and BPT. Although the occurrence of these disorders in our patient is most likely fortuitous, the possibility of an underlying channelopathy cannot be excluded. The role of channelopathies in the clinical expression of Williams syndrome should be further investigated.
